# Femoral Occlusion during Neonatal Cardiopulmonary Resuscitation Improves Outcomes in an Ovine Model of Perinatal Cardiac Arrest

**DOI:** 10.3390/children10111804

**Published:** 2023-11-13

**Authors:** Munmun Rawat, Srinivasan Mani, Sylvia F. Gugino, Carmon Koenigsknecht, Justin Helman, Lori Nielsen, Jayasree Nair, Upender Munshi, Praveen Chandrasekharan, Satyan Lakshminrusimha

**Affiliations:** 1 Department of Pediatrics, University at Buffalo, Buffalo, NY 14203, USA; sfgugino@buffalo.edu (S.F.G.); pkchandr@buffalo.edu (P.C.); 2 Department of Pediatrics, University of Toledo, Toledo, OH 43606, USA; 3 Department of Pediatrics, Weill Cornell Medicine, New York, NY 10065, USA; 4 Department of Pediatrics, Albany Medical College, Albany, NY 12208, USA; munshiu@amc.edu; 5 Department of Pediatrics, UC Davis Medical Center, Sacramento, CA 95817, USA; slakshmi@ucdavis.edu

**Keywords:** femoral occlusion, neonatal resuscitation, chest compression

## Abstract

Background: The goal of chest compressions during neonatal resuscitation is to increase cerebral and coronary blood flow leading to the return of spontaneous circulation (ROSC). During chest compressions, bilateral femoral occlusion may increase afterload and promote carotid and coronary flow, an effect similar to epinephrine. Our objectives were to determine the impact of bilateral femoral occlusion during chest compressions on the incidence and timing of ROSC and hemodynamics. Methodology: In this randomized study, 19 term fetal lambs in cardiac arrest were resuscitated based on the Neonatal Resuscitation Program guidelines and randomized into two groups: femoral occlusion or controls. Bilateral femoral arteries were occluded by applying pressure using two fingers during chest compressions. Results: Seventy percent (7/10) of the lambs in the femoral occlusion group achieved ROSC in 5 ± 2 min and three lambs (30%) did not receive epinephrine. ROSC was achieved in 44% (4/9) of the controls in 13 ± 6 min and all lambs received epinephrine. The femoral occlusion group had higher diastolic blood pressures, carotid and coronary blood flow. Conclusion: Femoral occlusion resulted in faster and higher incidence of ROSC, most likely due to attaining increased diastolic pressures, coronary and carotid flow. This is a low-tech intervention that can be easily adapted in resource limited settings, with the potential to improve survival and neurodevelopmental outcomes.

## 1. Introduction

Newborns who fail to respond to effective ventilation and chest compressions have a low survival rate, with a high incidence of neurodevelopmental disability among survivors [1,2]. In these infants, the time to the return of spontaneous circulation (ROSC) is an important prognostic indicator of the outcome [3]. The goal of chest compressions is to optimize cerebral and coronary perfusion leading to ROSC. Diastolic blood pressure is an important determinant of coronary perfusion pressure. Newly born infants requiring resuscitation in the delivery room have low diastolic pressure, due to the presence of circulatory collapse and left-to-right shunt through the patent ductus arteriosus (PDA) [4].

In the past, adult cardiopulmonary resuscitation (CPR) guidelines have recommended elevation of the lower extremities as it may promote venous return, cardiac preload, stroke volume, and augment artificial circulation [5,6]. It also increases the resistance to blood flow and shifts fluid from the lower extremities to the central circulation [7,8]. This approach has not been previously studied in neonates.

Hemodynamic effects similar to elevation of the lower extremities can be achieved through bilateral femoral occlusion in a neonate (including flexing of the lower limbs over the trunk, similar to the management of a hypercyanotic spell with tetralogy of Fallot). This maneuver can potentially increase the afterload to promote carotid and coronary flow, resulting in higher incidence and quicker ROSC. Additionally, it may act as a rapid intravenous volume expander by shifting blood from the lower extremities towards the intra-thoracic compartment (increasing the preload). Thus, the restricted cardiac output that is generated through CPR can be preferentially directed to the brain and the heart, resulting in higher incidence and quicker ROSC. This hemodynamic effect may be similar to that achieved through the administration of epinephrine and/or increasing the volume through the umbilical vein in the delivery room [9]. A major advantage of the femoral occlusion technique is that it can be performed rapidly within a few seconds of the initiation of chest compressions. Globally, it can be applied in low-resource settings and requires less skill and resources than placing an umbilical venous catheter and administering epinephrine.

## 2. Methods

The Institutional Animal Care and Use Committee at the State University of New York at Buffalo approved this study.

### 2.1. Animal Preparation

All procedures were consistent with the ARRIVE guidelines [10] and were approved by the Institutional Animal Care and Use Committee at the State University of New York at Buffalo.

Pregnant sheep (139–141 days gestation) were obtained from May Family Enterprises (Buffalo Mills, PA, USA). The ewes were sedated with intravenous diazepam (0.25–1.5 mg/kg) and ketamine (4 mg/kg) and anesthetized with 2–3% isoflurane. They were placed in a supine position and mechanically ventilated with 21% oxygen. Pulse oximetry and end-tidal carbon dioxide (EtCO_2_) were continuously monitored. A midline abdominal incision was made and the fetus within the uterus was partially exteriorized. The lamb was intubated with a 3.5–4.5 mm cuffed ETT and instrumented as previously described [11,12,13]. Lung liquid was allowed to drain due to gravity and the ETT was occluded to prevent gas exchange during the asphyxial phase of the experiment.

Intravenous fluids and medication were administered through a 16 g catheter placed in the jugular vein. Blood was sampled from an 18 g catheter in the carotid artery. Flow probes (Transonic Systems Inc., Ithaca NY, USA) were placed on the left carotid artery (2 mm), left pulmonary artery (4 mm), and left circumflex coronary artery (2 mm). The umbilical cord was cut, and an 18 g catheter was placed into the umbilical vein, 2–3 cm below the skin, for the administration of epinephrine. A 20 g catheter was placed into the umbilical artery for invasive blood pressure recording. A three-lead electrocardiogram was obtained (ECG100C; Biopac Systems, Inc., Goleta, CA, USA) and used Acknowledge Software Version 5.0 to record tracings from leads I, II, and III, the invasive pressure, and the rate of blood flow. Cardiac arrest secondary to asphyxia was defined by cessation of the carotid blood flow, arterial blood pressure, and auscultation confirming asystole.

### 2.2. Experimental Protocol

The lambs were randomized into the femoral occlusion and control group using sealed opaque envelopes. Twins were individually randomized. Due to the nature of the intervention, blinding was not possible. To decrease the chance of ROSC with PPV alone, resuscitation was started after five minutes of cardiac arrest with a T-piece resuscitator, using 21% O_2_, at a rate of 40 breaths/min and initial pressures of 35/5 cm H_2_O. The peak inflation pressure (PIP) was adjusted to obtain an adequate chest rise. After ventilation for 30 s, chest compressions were initiated.

**Figure 1:**
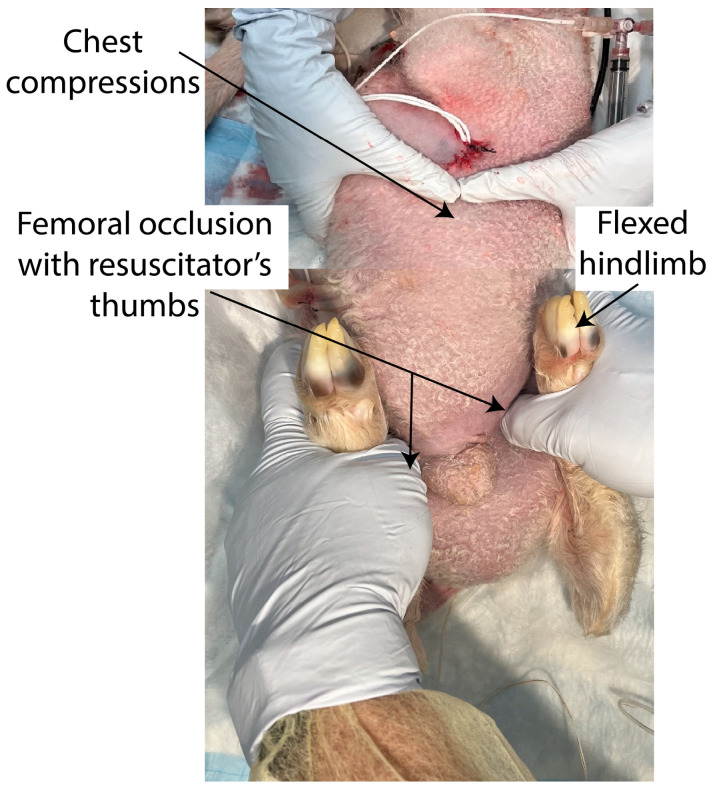
In the femoral occlusion group, during positive pressure ventilation and chest compressions, bilateral femoral arteries were continuously occluded by the resuscitator’s thumbs and flexion of hip joint until the return of spontaneous circulation or 20 min of cardiopulmonary resuscitation.

In the study group, the bilateral femoral arteries were continuously occluded using the resuscitator’s thumbs and hip flexion until the ROSC or 20 min had passed, whichever was earlier. The same resuscitator occluded the femoral arteries for all the experiments in this lamb model. The lamb was placed in the supine position and pressure was applied in the crease where the hind limbs join the anterior abdomen, midway between the pubic bone and the anterior superior iliac spine (Figure 1).

Upon the initiation of chest compressions, inspired O_2_ was increased to 100% as per the current American Heart Association (AHA) and Neonatal Resuscitation Program (NRP) recommendations [14,15]. If the return of spontaneous circulation (ROSC) was not achieved after 5 min, the first dose of epinephrine (0.03 mg/kg) was administered and flushed with 1 mL saline. Epinephrine was administered every 3 min thereafter, for a maximum of 4 doses or until the ROSC. The timing of the initial epinephrine dose was based on a simulation study that assessed the timing of the dose, the time to place an umbilical venous catheter (5–6 min), and an intubation time of less than 2 min [16].

### 2.3. ROSC

We defined the ROSC as a sustained heart rate > 100/min, with a systolic blood pressure > 30 mmHg. Resuscitation was stopped at 20 min if there was no ROSC, as per the current AHA and NRP recommendations [15,16]. ROSC was picked up by the rise in heart rate on the EKG and the systolic blood pressure on the Biopac machine. During this time, the CC were stopped and the ROSC was confirmed based on auscultation, the EKG monitor, the systolic blood pressure, and an increase in the flow and pressure in the carotid, coronary, and pulmonary arteries.

The lambs were placed on mechanical ventilation after achieving the ROSC. The FIO_2_ was adjusted by 5–10% every 30 s to maintain pre-ductal saturations between 85–95%. A tidal volume of 8 mL/kg was targeted by adjusting the PIP. The hemodynamic parameters were continuously recorded until approximately 2 h after birth. The lambs were then euthanized with 100 mg/kg of pentobarbital sodium (Fatal Plus^®^ Solution; Vortech Pharmaceuticals, Dearborn, MI, USA).

The plasma epinephrine levels were drawn from the jugular vein at the baseline, every 2 min during resuscitation, and after the ROSC. The levels were analyzed using commercially available ELISA kits (Epinephrine; Eagle Biosciences, Nashua, NY, USA). The reduced glutathione (GSH) to oxidized glutathione (GSSG) ratio in the plasma, as a marker of oxidative stress and reperfusion injury, was measured after derivatization with N-ethylmaleimide [17,18].

## 3. Data Analysis

Sample size estimation: Based on preliminary data, we needed nine lambs in each group to detect a difference of 180 s in the time to ROSC with femoral occlusion (standard deviation: 60 s), with a power of 80% and a type 1 probability of 0.05.

The statistics were evaluated using StatView 4.0 software. Continuous variables were analyzed using a two-way repeated measures ANOVA between the groups, and with Fisher’s post hoc test within the groups. The results are expressed as the mean and standard deviation. The chi-square test or Fisher’s exact test were used to evaluate the categorical variables when appropriate. Statistical significance was defined as *p* < 0.05.

## 4. Results

In this prospective, randomized study, 19 term fetal lambs were asphyxiated by umbilical cord occlusion, resulting in severe asphyxia leading to cardiac arrest. Ten lambs were randomized to the femoral occlusion group and nine lambs to the control group.

The baseline characteristics of all the lambs are shown in Table 1. The groups were similar in gestational age, birth weight, sex distribution, multiplicity, carotid artery blood flow, coronary artery blood flow, blood pressure, arterial pH, PaO_2_, and lactate, prior to the start of the experimental protocol. The time to asystole was similar in both of the groups.

### 4.1. Return of Spontaneous Circulation

With femoral occlusion, seven out of ten lambs (70%) achieved ROSC, while four out of nine control lambs achieved ROSC (44%) (2-sided *p* = 0.37 using Fisher’s exact test). The average time to achieve ROSC was faster in the femoral occlusion group (5 ± 2 min), as compared to the controls (13 ± 6 min, *p* = 0.02).

### 4.2. Epinephrine Doses

In the femoral occlusion group, three out of ten lambs achieved ROSC without requiring epinephrine, while all the lambs in the control group required epinephrine. The average number of epinephrine doses needed in the femoral occlusion group was 1.9 ± 1.8 and 3.4 ± 1.1 in the controls (*p* = 0.034).

## 5. Hemodynamic Parameters

**Figure 2:**
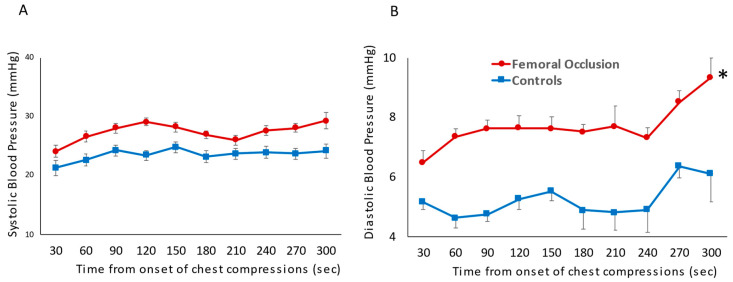
The following line graphs show blood pressures on the *y*-axis and time in seconds from the onset of chest compressions for the first 5 min prior to epinephrine administration on the *x*-axis. The red circles represent the femoral occlusion group and the blue squares represent the controls. Blood pressure measured invasively via pressure probe in the ascending aorta. Systolic blood pressure (**A**) and diastolic blood pressure (**B**) are shown at the time points prior to achieving ROSC and receiving the first dose of epinephrine (nine lambs in the controls at all time points, ten lambs in the study group 0–180 s, eight lambs in the study group at 210 and 240 s as two lambs achieved ROSC, and seven lambs at 270 and 300 s as a total of three lambs achieved ROSC prior to those time points) (* *p* < 0.05 using ANOVA repeated measures).

Blood Pressure: The systolic blood pressure was similar in the femoral occlusion (27.4 ± 1.5 mm Hg) and the control groups (23.5 ± 1 mm Hg) (Figure 2A). However, femoral occlusion resulted in higher diastolic pressures (7.7 ± 0.8 mm Hg) when compared to the controls (5.3 ± 0.6 mm Hg, *p* = 0.02) (Figure 2B). The mean blood pressure during the first three minutes was higher in the femoral occlusion group (13 ± 5 mm Hg), as compared to the controls (11 ± 3 mm Hg, *p* = 0.0003).

**Figure 3:**
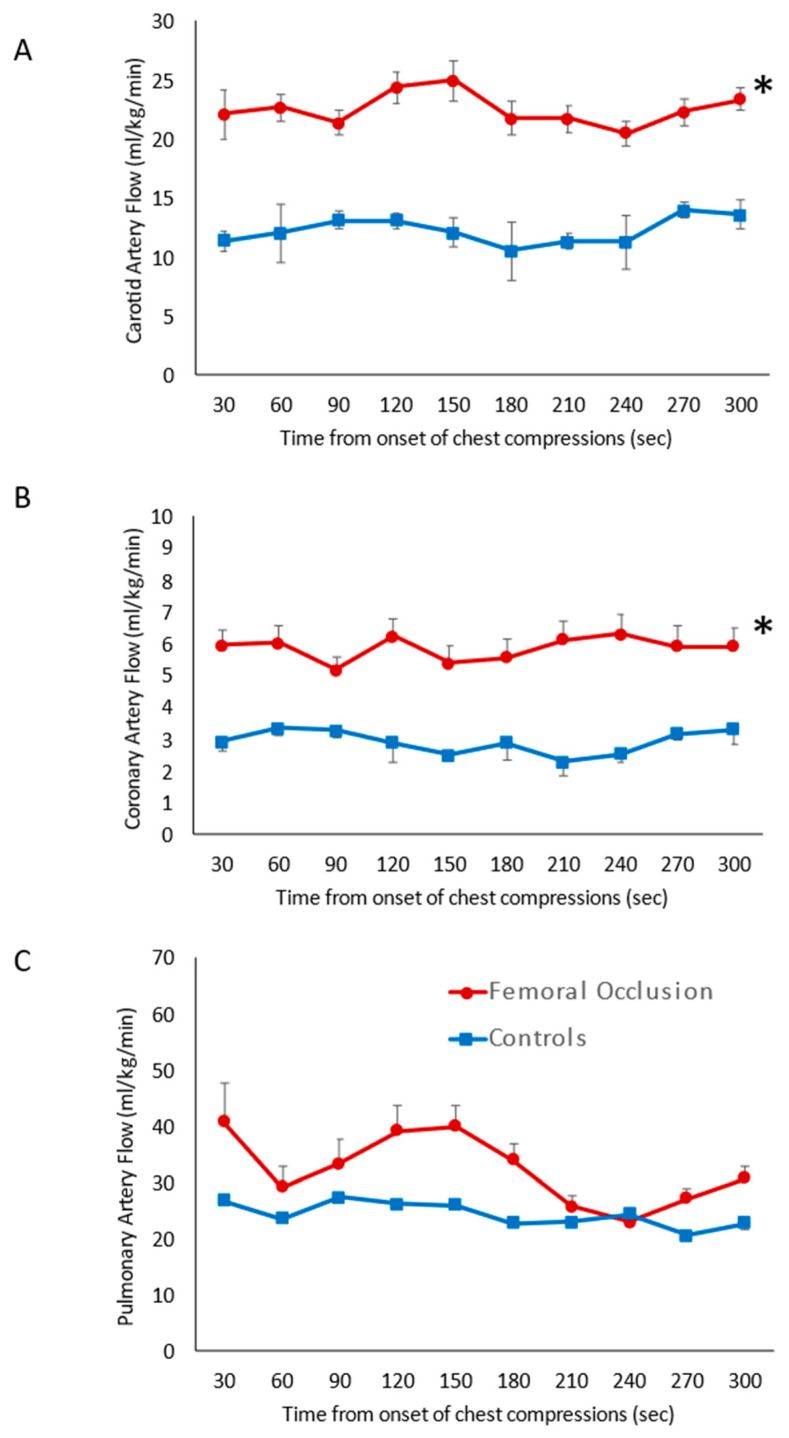
Systemic hemodynamics: the line graphs show blood flow on the *y*-axis and time in seconds from the onset of chest compressions for the first 5 min prior to epinephrine administration on the *x*-axis. The red circles represent the femoral occlusion group and the blue squares represent the controls. Data are represented as the average and standard error of the mean. (**A**) Maximal carotid blood flow (obtained from the left carotid artery); (**B**) maximal coronary artery flow (from left circumflex artery); and (**C**) maximal pulmonary blood flow (left pulmonary artery). The graphs represent data from nine lambs in the controls at all time points, ten lambs in the study group 0–180 s, eight lambs in the study group at 210 and 240 s as two lambs achieved ROSC, and seven lambs at 270 and 300 s as a total of three lambs achieved ROSC prior to those time points. * *p* < 0.05 using ANOVA repeated measures compared to the controls.

Carotid Artery Blood Flow (Figure 3A): During the first five minutes of resuscitation, prior to epinephrine administration, the average of the maximum carotid blood flow in the femoral occlusion group was higher at 22.6 ± 1.4 mL/kg/min when compared to the control lambs (12.3 ± 1.1 mL/kg/min, *p* = 0.001).

Coronary Artery Blood Flow (Figure 3B): Femoral occlusion during chest compressions resulted in higher coronary artery blood flow (5.9 ± 0.4 mL/kg/min) when compared to the control lambs that received only chest compressions (2.9 ± 0.4 mL/kg/min, *p* = 0.01) prior to the administration of epinephrine.

Pulmonary Artery Blood Flow (Figure 3C): No difference in the pulmonary blood flow was observed with femoral occlusion during chest compressions (32.4 ± 6.2 mL/kg/min) when compared to the control lambs (24.4 ± 2.1 mL/kg/min, *p* = 0.061). Flow across the ductus arteriosus was also similar between the two groups, 82 ± 8.5 mL/kg/min in the femoral occlusion group and 74 ± 3.4 mL/kg/min in the controls (*p* = 0.12).

In lambs that achieved ROSC, the femoral pulses were normal and there was no evidence of ischemia in the hind legs in all the lambs.

**Figure 4:**
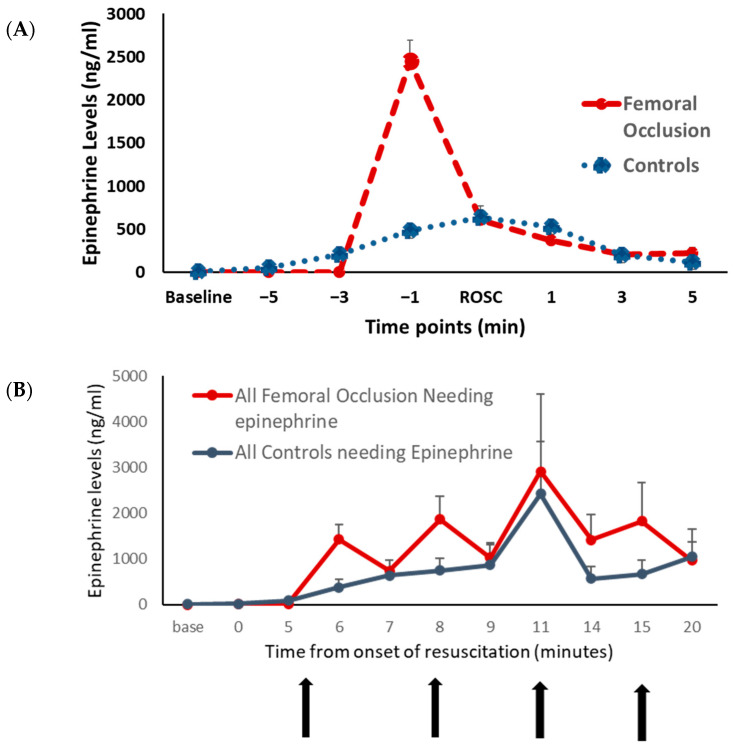
Plasma epinephrine level: the graphs show the plasma epinephrine levels at various time points. The plasma epinephrine levels in ng/mL are shown on the *y*-axis. The red circles represent the femoral occlusion group and the blue squares represent the controls. (**A**) Lambs that achieved ROSC are included. The *x*-axis shows time points in minutes prior to the return of spontaneous circulation (ROSC) and after ROSC. Femoral occlusion resulted in higher plasma epinephrine levels right before the ROSC. (**B**) All the lambs that received epinephrine are included. *x*-axis shows the time in minutes from the onset of resuscitation. Vertical arrows show the time points at which epinephrine was administered.

Epinephrine Levels (Figure 4): The plasma epinephrine levels were similar at the baseline between the two groups. In the lambs that achieved ROSC, just prior to the ROSC, the epinephrine levels were higher in the femoral occlusion lambs (2453 ± 878 ng/mL) as compared to the controls (482 ± 105 ng/mL, *p* = 0.04) (Figure 4A). Upon achieving ROSC, femoral occlusion was released, and the epinephrine levels were similar to the controls. In all the lambs that required epinephrine with and without ROSC, there was no significant difference in the epinephrine levels, most likely as the femoral occlusion group received fewer doses of exogenous epinephrine (Figure 4B).

**Figure 5:**
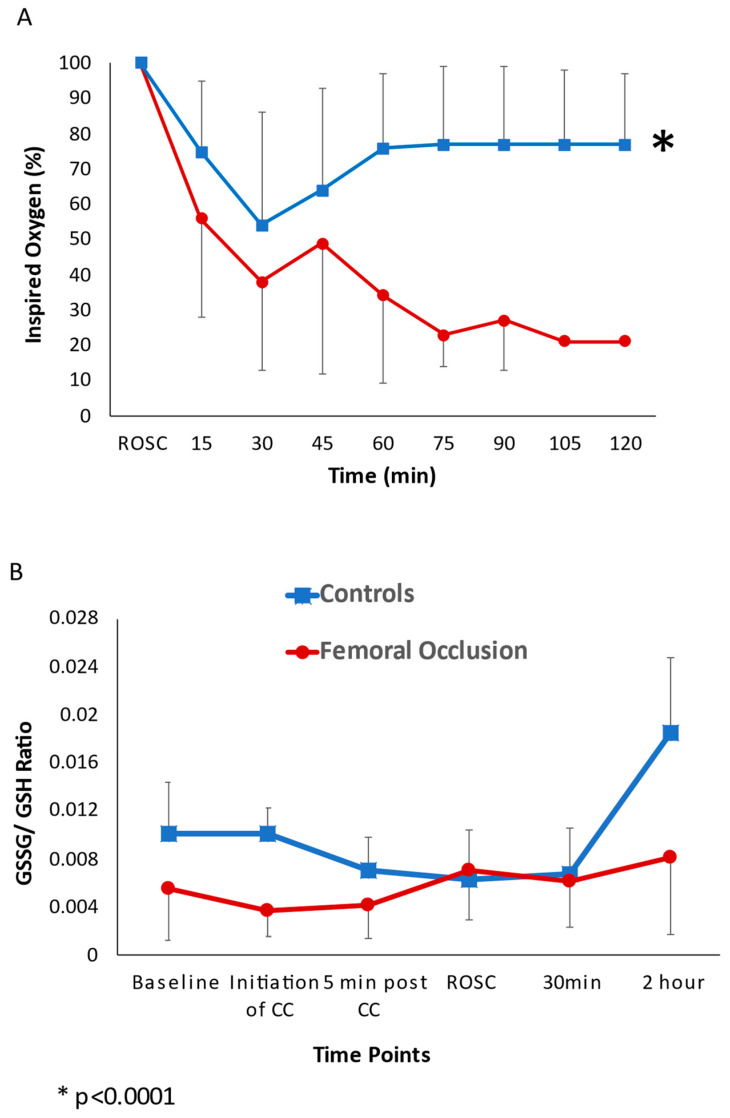
Oxygen exposure and oxidative stress: the line graphs show the inspired oxygen requirements and oxidative stress. The red circles represent the femoral occlusion group and the blue squares represent the controls. (**A**) Inspired oxygen requirements to maintain saturations as per the Neonatal Resuscitation Program guidelines. The *y*-axis shows the inspired oxygen (%) and *x*-axis shows the time in minutes post the return of spontaneous circulation. (**B**) Reduced glutathione (GSH) to oxidized glutathione (GSSG) ratio in plasma. Baseline GSSG/GSH ratios and ratios during chest compressions were similar between the two groups. At the completion of the study after 2 h, the GSSG/GSH ratio in the femoral occlusion group was 0.008 ± 0.006 and in the control lambs it was 0.016 ± 0.011, (*p* = 0.067).

Oxygen Exposure and Oxidative Stress (Figure 5): The duration of the exposure to 100% oxygen during chest compressions was shorter in the femoral occlusion group, as the time to ROSC was faster when compared to the controls. The average inspired oxygen needed, from the onset of chest compressions to the end of the study at 2 h after ROSC or at 20 min if no ROSC occurred, in the femoral occlusion group was 39 ± 29% and in the controls was 71 ± 35% (*p* < 0.0001). In the lambs that achieved ROSC, the average oxygen requirement from ROSC to the end of the experiment at 2 h in the femoral occlusion group was much lower (35 ± 25%) than the controls (56 ± 35%, *p* < 0.0001) (Figure 4A). The baseline GSSG/GSH ratios and the ratios during chest compressions were similar between the two groups. At the completion of the study after 2 h, the GSSG/GSH ratio in the femoral occlusion group was 0.008 ± 0.006 and in the control lambs it was 0.016 ± 0.011, *p* = 0.067 (Figure 4B).

## 6. Discussion

**Figure 6:**
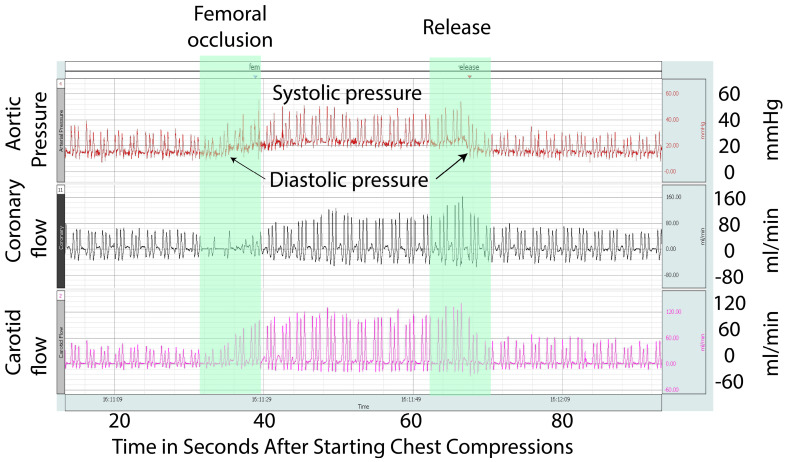
This is a Biopac^®^ recording snapshot from an asphyxiated lamb in asystole during chest compressions and PPV. This snapshot shows the aortic pressure, coronary artery flow, and carotid artery flow. Initiation of femoral occlusion is depicted by the green arrow and release by the red arrow. The picture shows an increase in the systemic pressures, including diastolic pressures, coronary artery blood flow, and carotid artery blood flow, from the baseline during the period of femoral occlusion.

This is the first study evaluating the effect of femoral occlusion in neonatal cardiopulmonary resuscitation. In our study, bilateral femoral occlusion during chest compressions resulted in faster time to the ROSC. We observed higher diastolic pressure and increased carotid and coronary blood flow with femoral occlusion (Figure 5 and Figure 6). The goal of chest compressions is to increase the coronary perfusion pressure (aortic diastolic pressure, right atrial pressure) and blood flow to the vital organs [19]. We speculate that femoral occlusion during chest compressions prevented the run-off of the already reduced cardiac output through the peripheral circulation and increased afterload. This in turn diverted the blood towards the heart, thus increasing coronary perfusion resulting in increased incidence and a quicker ROSC. Additionally, femoral occlusion resulted in a higher diastolic pressure, which may have resulted in improved coronary perfusion. Finally, a reduced volume of distribution of epinephrine due to femoral occlusion may have resulted in a higher pre-ROSC peak.

The blood flow and coronary perfusion during cardiac arrest are dependent on the quality and continuity of chest compressions [20]. The optimization of resuscitation interventions aims mainly at the rapid and effective rise in the coronary perfusion pressure (CPP) [21]. A CPP above 15 mm Hg appears to be necessary for the ROSC in adults [22]. However, the threshold of CPP is unknown in neonates due to the presence of PDA [4]. The diastolic blood pressure is an important determinant of coronary perfusion pressure and, in our study, we observed increased diastolic pressure with femoral occlusion. Similarly, an increase in the mean blood pressure may contribute to higher cerebral blood flow.

Resuscitative endovascular balloon occlusion of the aorta has been proposed as a novel approach to managing non-traumatic cardiac arrest in adults [23]. In a swine model of open chest resuscitation, percutaneous trans-femoral balloon aortic occlusion significantly increased the myocardial and cerebral perfusion [24]. Similarly, we also observed higher coronary and carotid flow with femoral occlusion. In another study using an adult porcine model with ventricular fibrillation, a passive leg raise by 45° during CPR increased the coronary perfusion pressure improving the ROSC rates and 24 h survival [25]. However, no studies have been conducted using a transitional neonatal model, such as fetal lambs evaluated in the current study.

We also wanted to see the effect of femoral occlusion on epinephrine levels. We anticipated that a reduced volume of distribution (Vd) due to femoral occlusion would result in a higher epinephrine concentration just prior to ROSC. In addition, we speculate that adrenals, being an essential organ, receive increased blood flow with femoral occlusion during chest compressions. This may also result in an inherent epinephrine surge. As expected, the epinephrine levels right before ROSC were higher in femoral occlusion.

Reduced glutathione (GSH) is considered to be one of the most important scavengers of reactive oxygen species (ROS), and its ratio with oxidized glutathione (GSSG) in the plasma may be used as a marker of oxidative stress and reperfusion injury [26]. Femoral occlusion showed a tendency towards reduced oxidative stress. We can attribute this to faster ROSC and, hence, the decreased exposure to 100% inspired oxygen during chest compressions. Additionally, the need for much lower inspired oxygen requirements post-ROSC could have resulted in reduced oxidative stress with femoral occlusion.

AHA and NRP recommend the initiation of chest compressions if the neonate’s heart rate remains below 60 bpm after at least 30 s of effective PPV [27]. Despite chest compressions and effective PPV, if the HR remains less than 60 bpm, NRP recommends administering epinephrine via a low lying umbilical venous catheter [17]. Epinephrine results in peripheral vasoconstriction, leading to increased diastolic pressure resulting in improved coronary perfusion [10]. However, the administration of epinephrine is a complex process that involves umbilical venous catheterization and the preparation of epinephrine injections. Elevation or folding of the lower extremities over the trunk or bilateral femoral occlusion in a neonate may act similar to epinephrine, increasing the afterload to promote coronary and carotid blood flow. Hence, folding the neonate’s lower extremities over the trunk or bilateral femoral occlusion using the resuscitator’s thumbs during chest compressions, while preparing for the intravenous administration of epinephrine, requires evaluation in a clinical setting. Femoral occlusion may be a more effective strategy than the administration of endotracheal epinephrine, while preparing for umbilical venous catheterization.

**Figure 7:**
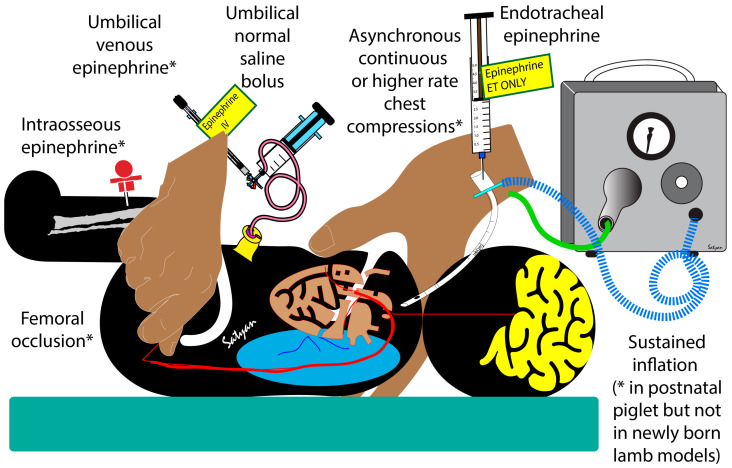
Strategies to increase diastolic pressure or cardiac output during chest compressions. * Indicates interventions that are effective in animal models (either newly born lambs or piglets). Umbilical venous or intraosseous epinephrine, femoral occlusion, and a higher rate of chest compressions are effective in increasing diastolic pressure or carotid flow. Sustained inflation increases diastolic flow in postnatal piglets, but not in newly born lambs.

Increasing the coronary perfusion pressure is a critical step in achieving ROSC and there are a number of strategies to increase diastolic pressure or cardiac output during chest compressions in neonatal resuscitation, but not all of them have been studied in humans. Increasing the diastolic pressure during chest compressions is most effectively achieved through umbilical venous (or intraosseous) epinephrine followed by a flush [4,28,29] (Figure 7). Sustained inflation increases the diastolic pressure in postnatal piglet models (possibly with a closed ductus) during chest compressions [30]. However, newly born lambs with an open ductus in cardiac arrest showed a decrease in diastolic pressure with sustained inflation during continuous chest compressions [31]. Endotracheal or intramuscular epinephrine is not associated with any significant hemodynamic improvement during chest compressions, possibly due to poor bioavailability [32,33]. O’Reilly et al. have shown increased stroke volume, but no improvement in diastolic pressure during chest compressions using a mechanical device [34]. Administering a normal saline bolus can potentially increase the diastolic pressure, but requires umbilical venous catheterization and intraosseous line placement and may be associated with pulmonary edema [35]. Continuous chest compressions asynchronous with ventilation at a higher rate may also be associated with increased diastolic pressure, or mean airway pressure, or carotid flow, due to the stair-step effect [36]. In the current study, we have shown that bilateral femoral occlusion during chest compressions is an effective and quick intervention to increase the diastolic pressure and requires minimal technical expertise or equipment.

Species difference was a limitation to our study, but the decision to use fetal lambs is based on their size in relation to the human neonate, and the comparable pulmonary physiology. Due to the technique involved, the resuscitators were not blinded and this might have introduced bias. We speculate that given the thin hind legs in fetal lambs, a human neonate may be a better model to evaluate lower limb flexion over the abdomen.

The major advantages of the femoral occlusion technique are that it can be performed quickly, is low tech, and can be used in a low-resource setting.

## 7. Conclusions

This is the first study evaluating the effect of femoral occlusion in a transitional model of cardiac arrest due to severe asphyxia. Femoral occlusion is a simple maneuver that can be easily adapted during CPR in resource-poor settings. We speculate the increase in diastolic pressure during femoral occlusion increases the coronary perfusion resulting in a faster ROSC. The results from this study are likely to trigger clinical trials that could change the neonatal resuscitation guidelines and significantly impact survival and neurodevelopmental outcomes for approximately 2400 neonates in the US and over 75,000 births worldwide that require extensive resuscitation in the delivery room [37,38].

## Figures and Tables

**Table 1:** Baseline characteristics.

Characteristics	Controls	Femoral Occlusion	*p*-Value
	(*n* = 9)	(*n* = 10)	
Gestational age (days)	141 ± 1	141 ± 0.8	0.3
Weight (kg)	4.4 ± 1.4	4.5 ± 0.8	0.8
Sex (Female %)	67	70	0.9
Multiplicity (%)	83	67	0.1
Carotid artery flow (mL/kg/min)	45 ± 20	50 ± 16	0.6
Coronary artery flow (mL/kg/min)	12 ± 11	11 ± 3	0.8
Diastolic blood pressure (mm Hg)	30 ± 11	31 ± 18	0.9
Time to asystole (min)	11.8 ± 10	10.2 ± 5	0.6
pH at asystole	6.8 ± 0.1	6.9 ± 0.1	0.1
PaO_2_ (mm Hg) at asystole	10 ± 2	11 ± 3	0.1
Lactate at asystole (mmol/L)	12 ± 3	11 ± 3	0.9

## Data Availability

Data presented in this manuscript will be available after complete analysis (2 h data) is conducted on request.
